# Anti-Disturbance Sliding Mode Control of a Novel Variable Stiffness Actuator for the Rehabilitation of Neurologically Disabled Patients

**DOI:** 10.3389/frobt.2022.864684

**Published:** 2022-05-02

**Authors:** Lufan Mo, Pengbo Feng, Yixin Shao, Di Shi, Linhang Ju, Wuxiang Zhang, Xilun Ding

**Affiliations:** ^1^ School of Mechanical Engineering and Automation, Beihang University, Beijing, China; ^2^ Beihang Goer (WeiFang) Intelligent Robot Co., Ltd, Weifang, China; ^3^ Beijing Advanced Innovation Centre for Biomedical Engineering, Beihang University, Beijing, China

**Keywords:** neurological disorders, rehabilitation exoskeleton robot, variable stiffness actuator, sliding mode control, impedance control

## Abstract

Lower limb exoskeletons are widely used for rehabilitation training of patients suffering from neurological disorders. To improve the human–robot interaction performance, series elastic actuators (SEAs) with low output impedance have been developed. However, the adaptability and control performance are limited by the constant spring stiffness used in current SEAs. In this study, a novel load-adaptive variable stiffness actuator (LaVSA) is used to design an ankle exoskeleton. To overcome the problems of the LaVSA with a larger mechanical gap and more complex dynamic model, a sliding mode controller based on a disturbance observer is proposed. During the interaction process, due to the passive joints at the load side of the ankle exoskeleton, the dynamic parameters on the load side of the ankle exoskeleton will change continuously. To avoid this problem, the designed controller treats it and the model error as a disturbance and observes it with the disturbance observer (DOB) in real time. The first-order derivative of the disturbance set is treated as a bounded value. Subsequently, the parameter adaptive law is used to find the upper bound of the observation error and make corresponding compensation in the control law. On these bases, a sliding mode controller based on a disturbance observer is designed, and Lyapunov stability analysis is given. Finally, simulation and experimental verification are performed. The wearing experiment shows that the resistance torque suffered by humans under human–robot interaction is lower than 120 *Nmm*, which confirms that the controller can realize zero-impedance control of the designed ankle exoskeleton.

## Introduction

Neurological disorders, such as stroke, Perkins syndrome, and cerebral apoplexy, can lead to long-term loss of motor function or even paralysis (WHO, 2006), which is undoubtedly a type of torture for patients. In response to this problem, research on rehabilitation of lower limb exoskeleton robots, such as BLEEX (Kazerooni et al., 2006), ALEX (Banala et al., 2010), Rewalk (Zeilig et al., 2012), Ekso (Pransky, 2014), and HEXAR (Kim et al., 2014), has gradually become a hot topic. Safe physical human–robot interaction (pHRI) is an essential characteristic of exoskeleton robots (De Santis et al., 2008; Falzarano et al., 2019; Shi et al., 2019). Thus, serial elastic actuators (SEAs) with compliance were introduced (Pratt and Williamson, 1995). However, a significant disadvantage of SEAs is that the stiffness is constant, which limits the energy storage capacity and bandwidth of the actuator (Kim et al., 2014). Therefore, a variable stiffness actuator (VSA) was designed as an improvement of the SEAs. Generally, VSA adopts two motors, one for controlling the stiffness and another for controlling the motion of the load side (Jafari et al., 2011; Wolf et al., 2011; Shao et al., 2021a). This makes the stiffness adjustment more flexible. For example, in the work of Shao et al. (2021a), it was proposed that the effective length of the force arm can be adjusted by the stiffness motor adjusting the contact point of the fulcrum on the lever to obtain variable stiffness. The stiffness motor is not affected by the load, which can greatly save energy compared to the VSA using the antagonistic principle (Popov et al., 2014). However, to achieve the lightweight and compact exoskeleton robot, the use of two motors is not a good choice. As an alternative, some scholars have studied non-linear variable stiffness actuators, which use only one motor (Thorson and Caldwell, 2011; Yu et al., 2013; Schepelmann et al., 2014; Shao et al., 2019; Hu et al., 2020; Shao et al., 2021b). Usually, human-like characteristics (small torque and low stiffness and large torque and large stiffness) are considered in non-linear VSA (Zhu et al., 2021). In the work of Thorson and Caldwell (2011), planetary gear mechanisms were introduced to obtain non-linear characteristics. In the work of Hu et al. (2020), multiple symmetric cams were introduced to bidirectional non-linear characteristics. Both methods increase the size of the actuator. In the work of Shao et al. (2021b), a compact load-adaptive variable stiffness actuator (LaVSA) was designed, which was inspired by the structure of the human ankle joint. LaVSA can customize the cam profile to alter the stiffness curve and be more energy efficient, lighter, and more compact than SEAs. However, as discussed in *Preliminary*, these advantages of LaVSA also bring complexity at the mechanical level and larger mechanical clearance and friction, which make the controller more robust.

Due to the introduction of variable stiffness, there is usually a larger gap in the VSA system. The machining accuracy makes it difficult to ensure that the stiffness curve achieves the expected effect. In addition, variable stiffness will result in non-linear problems such as backlash, hysteresis, and dead zones (Zhao et al., 2019). These will greatly increase the inaccuracy and chattering of the control. To overcome these problems, various control methods have been proposed in recent years. They are mainly divided into three categories: position control, force control, and force position hybrid control (Meng et al., 2015). Position-based tracking control is significant in the early rehabilitation phase when the impaired limb is unable to move. In the work of Sardellitti et al. (2012), a gain scheduling method based on a linear quadratic regulator (LQR) was proposed to obtain high-precision position control. This method can continuously adjust the control parameters according to the current stiffness of the flexible transmission. Then, in the work of Sardellitti et al. (2013), an improved controller based on LQR was proposed, and a more complete formula, experiment, and stability analysis were provided. In the work of Zhakatayev et al. (2015), a non-linear model predictive control (NMPC) algorithm was proposed to complete the hybrid control of position and speed on the load side. However, the controller requires high sensor performance and is sensitive to disturbances. Clearly, it is not suitable for ankle exoskeleton environments that are disturbed by human-robot interaction. In the work of Zhang et al. (2017), an adaptive neural network controller (NNC) based on DOB was proposed to control the joint angle of non-linear VSA. The controller shows strong robustness to disturbances, such as gap, hysteresis, and non-linearity, which are the difficulties faced in non-linear VSA control. When rehabilitation training is performed to a certain extent or the person has certain exercise ability, it should be considered that the person will take the initiative to exercise. Both force control and force position hybrid control take into account that problem. In the work of Simon et al. (2007), a hybrid position and force controller was designed to provide the target resistance force to the impaired limb to improve force symmetry in the limbs during lower limb extensions. In the work of Wang et al. (2016), a sliding mode control (SMC) strategy based on DOB was proposed. The input torque at the load side is controlled by controlling the deformation of the elastic element. To suppress the chatter problem of SMC Huang et al. (2008) introduced the saturation function instead of the symbolic function. To allow for natural variations in the patient, the robot’s position and contact force need to be adjusted in real time. The impedance control strategy is one of the most suitable force control methods. Liu et al. (2018) performed much work in this regard. A gain-scheduled torque controller that applies the Linear Quadratic Gaussian (LQG) technique to deal with the multiloop feedback in the VSA plant was proposed. An impedance control based on cascaded position-torque control loops (Liu et al., 2019) was proposed. The LQG approach is also employed here, but with additional discussion on the observer designed to serve the proposed cooperative control approach. On these bases, it is further proposed to use human joint torque as feedback and use the Lyapunov function to obtain the range of stiffness operation (Liu et al., 2021).

The compact, lightweight, and energy-efficient characteristics of LaVSA make it suitable for exoskeleton joints (Huo et al., 2014; Chen et al., 2021), especially ankle joint exoskeletons. As mentioned in *Preliminary*, LaVSA exhibits greater mechanical clearance and friction loss. Therefore, in this study, we designed a robust SMC based on DOB. Aiming at the irregular change of dynamic parameters at the load side of the ankle exoskeleton, the load-side dynamics and model errors are unified as disturbance, which is observed by DOB. The controller realistically treats the first-order derivative of the disturbance as a bounded value, compared to the work of Yu et al. (2015), which treats disturbance as constant. A saturation function is introduced instead of a sign function to suppress chattering. Taking into full account the initiative of people in rehabilitation training, as mentioned above, impedance control is a more commonly used method in rehabilitation exoskeletons. Then, we use DOB-based SMC for zero-impedance control of the ankle exoskeleton so that the robot can follow the movement of the patient, and the patient can move freely without much effort.

The remainder of this article is organized as follows. *Preliminary* presents a previously designed LaVSA prototype, focusing on the dynamic analysis and important improvements of the mechanical structure. *Controller Design* presents the design process of the anti-disturbance sliding mode controller for LaVSA. *Simulation and Experiment* presents the simulations and experiments, focusing on zero-impedance control. Finally, *Conclusion* presents the main conclusions.

## Preliminary

### System Setup

**FIGURE 1:**
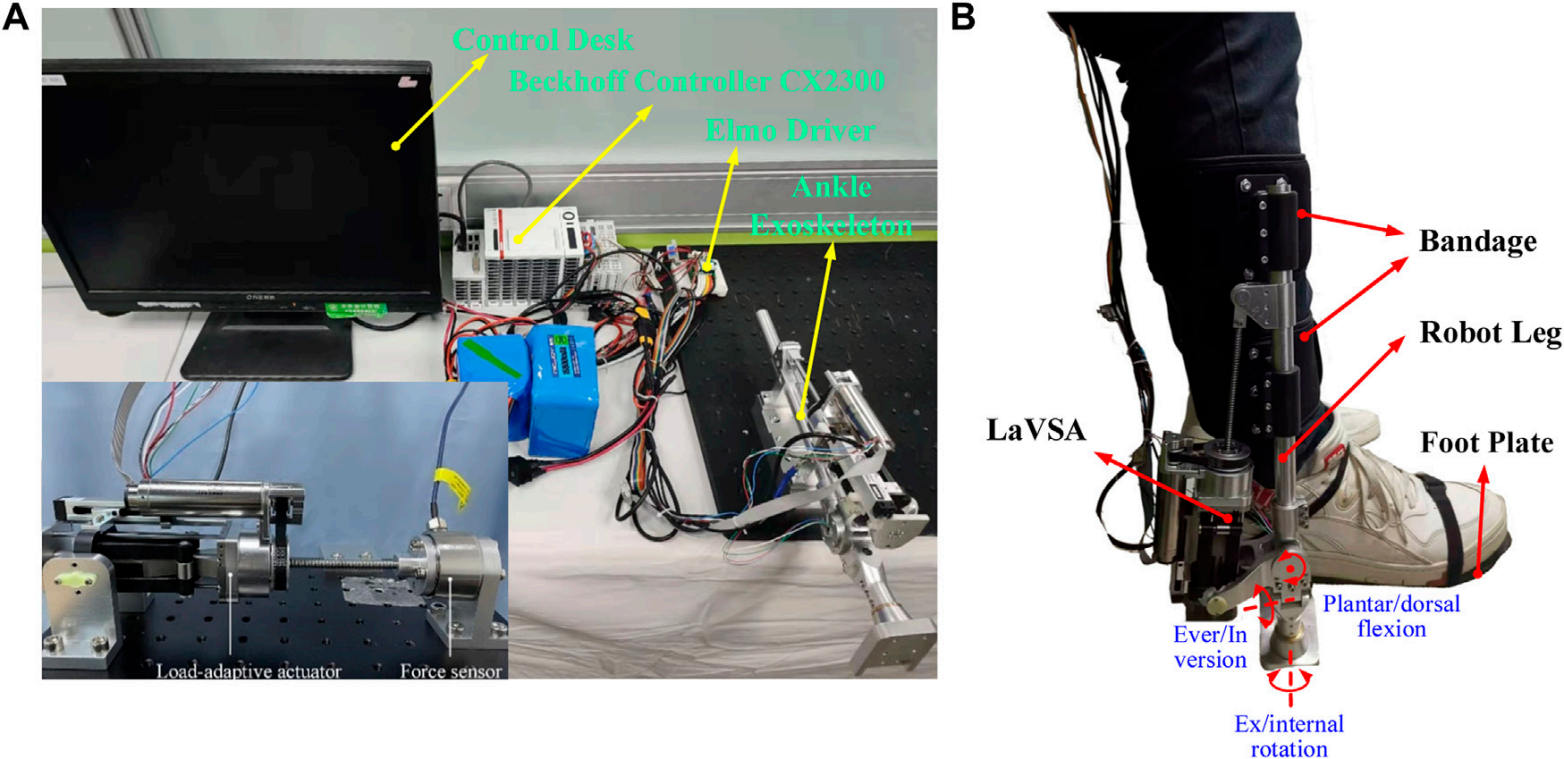
Schematic diagram of the experimental setup. **(A)** Testing platform. **(B)** Human–robot interaction scene.

**FIGURE 2:**
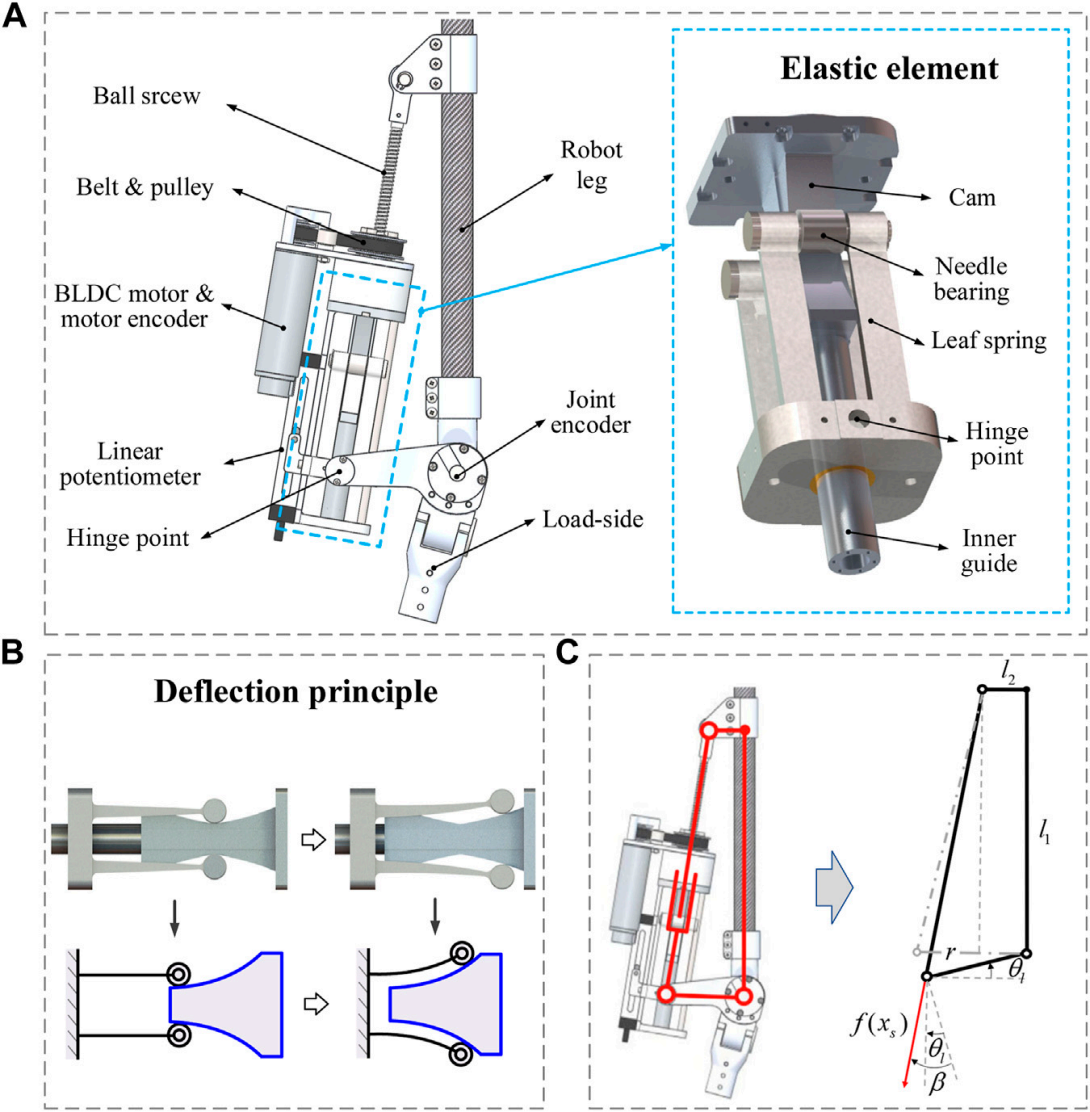
Load-adaptive variable stiffness actuator. **(A)** Components of the LaVSA model. **(B)** Leaf spring deflection principle. **(C)** Mechanism diagram of LaVSA.

The schematic diagram of the testing platform, shown in Figure 1A, displays the main system elements. The middle of the picture shows the stable and reliable Beckhoff controller (CX2300, sampling time of 1 ms), which uses the Enthercat protocol to communicate with the ControlDesk and Elmo Drivers. Through the TwinCat3 software installed in the ControlDesk, the real-time control and feedback signal of the ankle exoskeleton system are received. The right of the picture shows the test bench equipped with the ankle exoskeleton. For safety, the emergency stop switch is connected to the circuit. Details of the LaVSA mounted on the ankle exoskeleton are shown in Figure 2A. The actuator is equipped with a Maxon EC-4pole 120 W DC servo motor, a Maxon ENX16 EASY incremental encoder, and a non-linear stiffness mechanism/elastic element, as well as an RLS RM08 12-bit absolute used to measure the joint angle. The relative displacement relative to the screw between the leaf spring and cam, which is treated as a deflection in this paper, is measured by a Miran KSF-35 linear potentiometer. The human–robot interaction scene of the ankle exoskeleton is shown in Figure 1B. The ankle joint of the human body and the ankle exoskeleton are approximately on the same axis to control the person’s plantar/dorsal flexion; ever/inversion and ex/internal rotation movements of the ankle exoskeleton are passive. Under the action of the bandage and footplate, the robot leg and the human leg are relatively stationary.

LaVSA works as follows. The torque generated by a servo motor drives a small pulley to rotate, and the small pulley drives the large pulley to rotate so that the actuator moves on the lead screw, which in turn drives the leaf spring to move. The leaf spring and the load are hinged. When the load increases, the relative displacement between the leaf spring and the cam increases, the deformation of the leaf spring increases (the deformation principle of leaf spring is shown in Figure 2B), and the elastic force generated by the leaf spring increases. Furthermore, the driving force received by the load increases. Consequently, the motion transition of the actuator can be divided into three parts. The first part is the rotary motion of the servo motor, the small pulley, and the large pulley. The second part is the translation of the actuator on the screw. The third part is the rotational movement of the load side. In contrast to most current VSAs, such as in Wang et al. (2015) and (Hu et al. (2020), the motion transfer is only rotation-to-rotation (rotation of motor side to rotation of load side). Such more complex motion transfer will inevitably result in greater mechanical clearance and friction loss, especially at the ball screw. Different stiffness curves can be obtained by designing different cam profiles. The cam profile we use in this article will allow the actuator to achieve low-load, low-stiffness, high-load, and high-stiffness characteristics that match the motion mechanism of the human body.

### Control Model

The actuator dynamic model can be divided into three parts. The first part is the rotary motion of the motor-side. The second part is the translation of the actuator. The third part is the rotary motion of the load-side. The dynamic models of these three parts are given as follows:

$$\tau_{m}=J_{m}{\ddot{\theta }}_{m}+b_{m}{\dot{\theta }}_{m}+\tau_{in}+d_{m},$$ (1)

$$F_{in}=m_{a}{\ddot{x}}_{a}+b_{a}{\dot{x}}_{a}+f\left(x_{s}\right)+d_{a},$$ (2)

$$f\left(x_{s}\right)r\cos\beta =J_{l}{\ddot{\theta }}_{l}+b_{l}{\dot{\theta }}_{l}+d_{l}.$$ (3)

The motor-side model is expressed using Eq. 1, where $\theta_{m}$ represents the motor output position; $\tau_{\text{m}}$ represents the torque of the motor; $J_{\text{m}},\;b_{\text{m}}$ represent the equivalent inertia and damping coefficients of two belt wheels and motor, respectively; $\tau_{in}$ represents the resistance torque equivalent to the motor-side; $d_{\text{m}}$ is the model inaccuracy and friction of the motor-side. The actuator model is expressed using Eq. 2, where $x_{a}$ and $x_{s}$ are the displacement of the actuator relative to the screw and the deflection of the elastic element, respectively; $F_{in}$ is the equivalent force of the active torque on the ball screw; $m_{a}$ and $b_{a}$ are the mass and damping coefficient of the actuator, respectively; $d_{a}$ is the model inaccuracy and friction of the actuator; and $f\left(x_{s}\right)$ is the reaction force generated by the elastic deflection during stiffness variation. The load-side model is expressed using Eq. 3, where $\theta_{l}$ is the load side angle position; r is the length of the link from the center of the joint to the elastic element; β is the included angle between the connecting rod vertical direction and lead screw direction; $J_{l}$ and $b_{l}$ are the inertia and damping coefficient of the load side, respectively; and $d_{l}$ is the model inaccuracy and friction of the load side.

$$\tau_{in}=F_{in}N,$$ (4)

where $N=\frac{p}{2\pi n}$ , in which p is the lead of the lead screw and n is the transmission ratio of the pulley.

The mechanism diagram of LaVSA is shown in Figure 2C. For the convenience of calculation, the position of the joint connecting rod perpendicular to the robot leg is regarded as the initial position, and the following relationship can be obtained:

$$\cos\beta =\frac{\left(r\sin\theta_{l}+l_{1}\right)\cos\theta_{l}-\left(r\cos\theta_{l}-l_{2}\right)\sin\theta_{l}}{\sqrt{{\left(r\cos\theta_{l}-l_{2}\right)}^{2}+{\left(r\sin\theta_{l}+l_{1}\right)}^{2}}},$$ (5)

$$x_{l}=\sqrt{{\left(r\cos\theta_{l}-l_{2}\right)}^{2}+{\left(r\sin\theta_{l}+l_{1}\right)}^{2}}-\sqrt{{\left(r-l_{2}\right)}^{2}+l_{1}^{2}},$$ (6)

$$x_{a}=x_{s}+x_{l}=N\theta_{m},$$ (7)

where $x_{l}$ is the displacement of the hinge point between the link and the elastic element on the link relative to the lead screw.

In the process of human-robot interaction, due to the presence of passive joints on the load side of the ankle exoskeleton, the load parameters $J_{l}$ will change continuously and irregularly. Model-based control is not possible. Therefore, the dynamic model without considering the load parameters is taken as the object, and the right side of Eq. 3 is unified as a disturbance. Then, the control model can be shaped as follows.

Combining Equations 1–7,

$$\tau_{m}=m{\ddot{x}}_{s}+b{\dot{x}}_{s}+Nf\left(x_{s}\right)+d,$$ (8)

where

$$\begin{array}{l} m=\frac{1}{N}J_{m}+Nm_{a},\\ b=\frac{1}{N}b_{m}+Nb_{a},\\ d=\left(\frac{J_{m}}{N}+Nm_{a}\right){\ddot{x}}_{l}+\left(\frac{b_{m}}{N}+Nb_{m}\right){\dot{x}}_{l}+Nd_{a}+d_{m}, \end{array}$$ (9)

where d is regarded as a disturbance set.

### Force-Deflection Curve Calibration

To obtain the large force range and stiffness characteristics, we re-optimized the cam curve to make the non-linearity of force-deflection more obvious. Additionally, it should be noted that the force of the elastic body cannot exceed the service limit. This is different from the work done in a previous study (Shao et al., 2021b).

Remark 2.1 The range of the elastic element force–deformation curve is selected as follows: force is 200–200 N, deformation is 7.5–7.5 mm; the peak torque that the elastic element can withstand is ${\left(200N\times r\times cos\beta \right)}_{\max}=12$ Nm, which is far greater than the peak torque of the human ankle joint of 2 Nm at a speed of 2.7 m/s (Witte et al., 2020). This means that this curve can be used in the ankle exoskeleton.

In regard to controlling the model of (8), the variable $f\left(x_{s}\right)$ must be known. Therefore, the force-deflection relationship between $f\left(x_{s}\right)$ and $x_{s}$ should be obtained. In this work, by fixing the load as shown in the lower-left corner of Figure 1A, the values of $F_{in}$ and $x_{s}$ can be measured by a Simbatouch SBT673 force sensor and Miran KSF-35 linear potentiometer. The motor moves at a constant speed until the elastic body produces a certain amount of deformation $x_{s}$ and then makes the reverse movement at the same speed to $-x_{s}$ . Of note, the values of $F_{in}$ and $f\left(x_{s}\right)$ are approximately equal in Eq. 3 when the speed is slow. However, the speed cannot be too small to cause the Coulomb friction to increase. Due to the accuracy of cam machining, the forward deformation and reverse deformation curves are different. To reduce the error, we use a piecewise function to fit the force-deflection curve according to the theoretical formula in a previous study (Shao et al., 2021b). The corresponding formula is

$$\left\{ \begin{array}{l} f\left(x_{s}\right)=sign\left(x_{s}\right)a_{p}\left(e^{b_{p}\left|x_{s}\right|}-1\right)\text{, }\left(x_{s}\text{>0}\right)\text{, }\\ f\left(x_{s}\right)=sign\left(x_{s}\right)a_{n}\left(e^{b_{n}\left|x_{s}\right|}-1\right)\text{, }\left(x_{s}\leq \text{0}\right). \end{array}\right.$$ (10)

**FIGURE 3:**
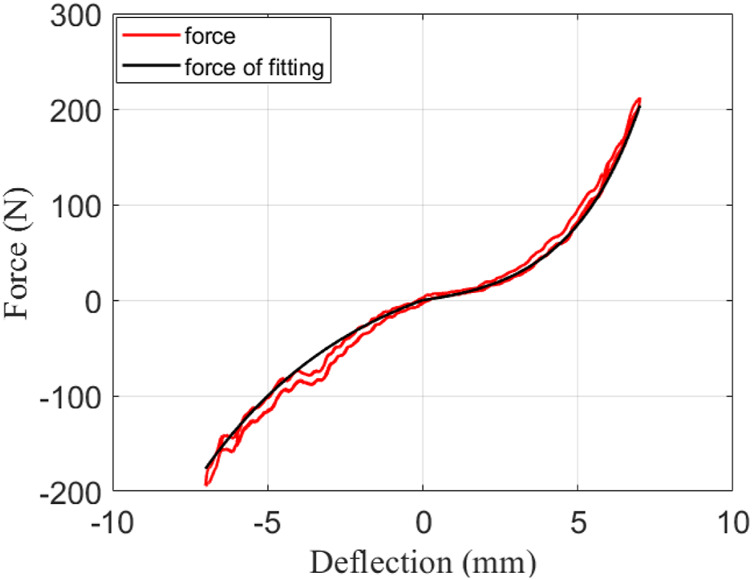
Force-deflection curve fitting diagram.

When ${\dot{x}}_{s}={\dot{x}}_{a}$ = 0.15 mm/s, experiments with $x_{s}=6$ mm, 6.5 mm, and 7 mm are performed to calibrate the force-deflection curve, as illustrated in Figure 3. The fitted parameters are shown in Table 1. It is observed that the fitting effect is very good. Compared to a previous study, the non-linearity of the curve is more obvious when the deformation amount is less than 7 mm. Of note, the fitted force and the actual force have a large error of approximately 26 N when $x_{s}=-3.5$ mm. This is because the surface machining (wire cutting) of the cam is not ideal. This error is solved by the controller design, which also determines that the designed controller must have a certain robustness.

**TABLE 1:** Fitted parameters.

Parameters	$a_{p}$	$b_{p}$	$a_{n}$	$b_{n}$
Value	10.06	0.4366	62.52	0.1915

## Controller Design

### Control Model

A closed-loop controller based on sliding mode control, which is aimed to improve the accuracy and robustness, was designed. The following specifications were considered to establish the impedance control model for the VSA system described in Eqs 1–3.

**FIGURE 4:**
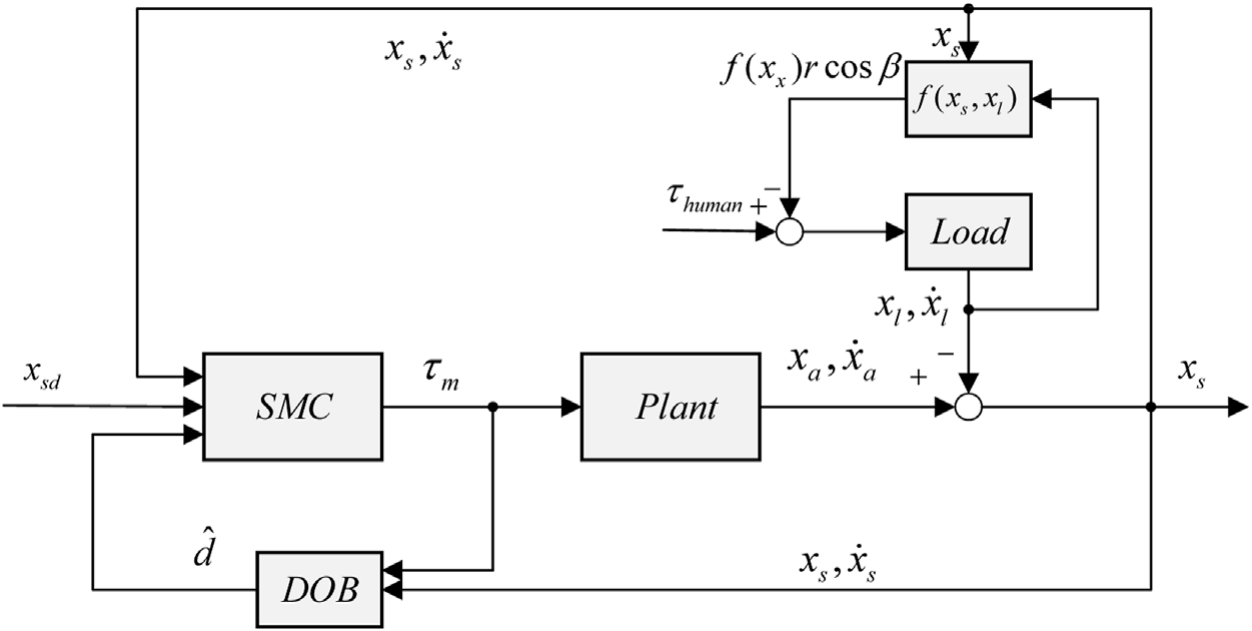
Zero-impedance control block diagram.

1) Fixed-output case: Fixing the output of the VSA is a suitable test case for the controller performance. In this case, the absence of the load side model of Eq. 3 leads to elastic deflection only with respect to the position of the motor, which means that there is no need to introduce an additional sensor to measure $x_{s}$ . Moreover, the disturbance set d is smaller, which means that the control parameters are easier to adjust. As a result, the elastic deflection is $x_{s}=x_{a}=N\theta_{m}$ , which means that $f\left(x_{s}\right)$ can be controlled by controlling $x_{a}$ . The corresponding relationship is $x_{ad}=x_{sd}\Leftrightarrow f{\left(x_{s}\right)}_{d}$ . The disturbance set is $d=Nd_{0}+d_{m}$ .
2) Zero-impedance control: To realize the active control of patients without much effort that can be regarded as zero-impedance control, the controller must perform torque control when the load side is not fixed. When the main driving force provided by humans drives the load to move, Eq. 3 can be expressed as

$$\tau_{human}-f\left(x_{s}\right)r\cos\beta =J_{l}{\ddot{\theta }}_{l}+b_{l}{\dot{\theta }}_{l}+d_{l}$$ (11)

where $\tau_{human}$ represents the torque on the load side that is acted on by the human. Of note, when the speed of joint ${\dot{\theta }}_{l}$ is constant, the right side of Eq. 11 is equal to the damping force $b_{l}{\dot{\theta }}_{l}$ . The value $b_{l}{\dot{\theta }}_{l}$ is actually so small that it can be ignored. Thus, the magnitude of the human torque can be measured by the elastic force. Zero-impedance control can be achieved by making the target deformation of the elastic body $x_{sd}=0$ . The corresponding control block diagram is shown in Figure 4.

### Torque Controller Design

In *Control Model*, the variation in load parameters, model error, and friction are regarded as a disturbance set. The handling of the disturbance set is an important part of the controller design. To reduce the sensitivity of the control performance to disturbance, DOB are designed to predict disturbance sets and are given as

$$\left\{ \begin{array}{l} \dot{z}=K\left(\tau_{m}-bx_{s}-Nf\left(x_{s}\right)\right)-K\hat{d},\\ \hat{d}=z+Km{\dot{x}}_{s}, \end{array}\right.$$ (12)

where $\hat{d}$ is the estimation of d , K is the observer gain, and z is an auxiliary variable. Then, the first derivative of the observation error can be calculated as

$$\begin{array}{l} \dot{\tilde{d}}=\dot{d}-\dot{\hat{d}}=\dot{d}-\dot{z}+Km{\ddot{x}}_{s},\\ \;=\dot{d}-K\left(\tau_{m}-bx_{s}-Nf\left(x_{s}\right)\right)-K\hat{d}+Km{\ddot{x}}_{s}S,\\ \;=\dot{d}-K\tilde{d}. \end{array}$$

By integrating both sides of the abovementioned equation, we obtain

$$\tilde{d}\left(t\right)=\tilde{d}\left(0\right)e^{-Kt}+e^{-Kt}\int_{0}^{t}\dot{d}\left(\tau \right)e^{K\tau }d\tau .$$ (13)

For the sake of simplicity, some observers are usually designed based on the assumption that $\dot{d}=0$ , which is inconsistent with the real situation, especially in the control system of the rehabilitation exoskeleton robot. This assumes that $\left|\dot{d}\right|\leq D_{1}$ is more realistic, where $D_{1}$ is an unknown disturbance. Therefore, Eq. 13 can be further simplified as

$$\begin{array}{l} \tilde{d}\left(t\right)\leq \tilde{d}\left(0\right)e^{-Kt}+e^{-Kt}D_{1}\int_{0}^{t}e^{K\tau }d\tau ,\\ \;=\tilde{d}\left(0\right)e^{-Kt}+\frac{D_{1}}{K}e^{-Kt}\left(e^{Kt}-1\right)=\tilde{d}\left(0\right)e^{-Kt}+\left(\frac{D_{1}}{K}-\frac{1}{e^{Kt}}\right). \end{array}$$ (14)

It is observed that the error of the observer is affected by $D_{1}$ and bounded. Let this upper bound be $\left|\tilde{d}\left(t\right)\right|\leq D$ . To facilitate the compensation of this error, it is necessary to design an upper bound adaptive law to find the estimated value $\hat{D}$ of D . The estimation error of the upper bound is defined as $\tilde{D}=D-\hat{D}$ . The adaptive law is as follows:

$$\dot{\hat{D}}=\gamma \left|s\right|,$$ (15)

where $\dot{\hat{D}}$ is the first derivative of the estimate of the upper bound D ; γ is the gain of the upper bound adaptive law, which is a positive constant, and s is the sliding surface defined to describe the error dynamics of $e=x_{s}-x_{sd}$ as follows:

$$s=ce+\dot{e}.$$ (16)

Finally, the sliding mode control law based on the disturbance observer is derived as follows:

$$\tau_{m}=b{\dot{x}}_{s}+Nf\left(x_{s}\right)+\hat{d}-k_{0}s-\hat{D}\mathrm{sgn}\left(s\right)+m{\ddot{x}}_{sd}-mc\dot{e},$$ (17)

where c and $k_{0}$ are positive constants, which can be regarded as the control gain, and sgn (.) is a standard sign function.

### Stability Analysis

To ensure the correctness of the controller design, the following Lyapunov function is considered:

$$V=\frac{1}{2}ms^{2}+\frac{1}{2}{\tilde{d}}^{2}+\frac{1}{\gamma }{\tilde{D}}^{2}.$$ (18)

Differentiating Eq. 18 and substituting Eqs 14–17 into it yields

$$\begin{array}{l} \dot{V}=ms\dot{s}+\tilde{d}\dot{\tilde{d}}+\frac{1}{\gamma }\tilde{D}\dot{\tilde{D}},\\ \;=-k_{0}s^{2}-\tilde{d}s-\hat{D}\left|s\right|+\tilde{d}\dot{\tilde{d}}-\tilde{D}\left|s\right|,\\ \;\leq -k_{0}s^{2}+D\left|s\right|-\hat{D}\left|s\right|-\tilde{D}\left|s\right|+\tilde{d}\dot{\tilde{d}},\\ \;=-k_{0}s^{2}+\tilde{d}\dot{\tilde{d}}. \end{array}$$ (19)

Integrating Eq. 19 yields

$$V\left(t\right)\leq V\left(0\right)-k_{0}\int_{0}^{t}s^{2}d\tau +\frac{1}{2}{\tilde{d}}^{2}.$$ (20)

Therefore, it can be obtained that

$$V\left(t\right)+k_{0}\int_{0}^{t}s^{2}d\tau \leq V\left(0\right)+\frac{1}{2}D^{2}.$$ (21)

Because D are bounded, combining Eq. 21 shows that $V\left(t\right),\;\int_{0}^{t}s^{2}d\tau ,\;s$ are bounded. Then, using Eq. 19, $e,\;\dot{e},\;\dot{\tilde{d}},\;\tilde{D},\;\dot{\tilde{D}}$ are bounded and can be obtained. Thus, $\dot{s}$ is bounded. In other words, s is uniformly continuous for time t . According to Barbalat’s lemma, it can be concluded that

$$\underset{t\to \infty }{\lim}s\to 0\Rightarrow \underset{t\to \infty }{\lim}e\to 0.$$

## Simulation and Experiment

In this section, the results of the simulation, experimental platform test, and wearable test are presented to verify the effectiveness of the controller. The corresponding parameters are shown in Table 2. Of note, to suppress the chatter of SMC, most scholars use the saturation function instead of the sign function. This study also applies a similar approach in simulations and experiments. Its essence is that outside the boundary layer, switching control is used to rapidly make the system state in a sliding mode. In the boundary layer, feedback control is used to reduce the chattering during fast switching of sliding modes. The saturation function is as follows:

$$sat\left(s\right)=\left\{ \begin{array}{l} 1,\text{ s>}\text{Δ},\\ \frac{1}{\text{Δ}}s,\;\left|s\right|\leq \text{Δ},\\ -1,\text{ s<}\text{Δ}. \end{array}\right.$$ (22)

**TABLE 2:** System parameter and mechanism size.

Parameters	m	b	r(mm)	$l_{1}\left(mm\right)$	$l_{2}\left(mm\right)$	n	p(mm)
Value	0.0521	6.1289	60	210	27	2.5	2

Remark 4.1 The choices of dimension parameters of the mechanism (such as r , $l_{1}$ , $l_{2}$ ) are based on the experience of the designer accumulated from trial and error in simulation studies, while the choices of dynamic parameters (such as m , b ) are based on parameter identification. In fact, there is no way to accurately identify the dynamic parameters of the system. In this study, the disturbance observer is used to observe this error in real time and make corresponding compensation in the control law.

Remark 4.2 In this study, the controller is designed by putting the dynamics of the load side into the disturbance set. Therefore, the dynamic parameters of the load side are not necessary.

### Simulation

For the two torque control modes mentioned in *Control Model*, two modes were simulated to verify the correctness of the theoretical analysis and provide a basis for parameter adjustment.

**FIGURE 5:**
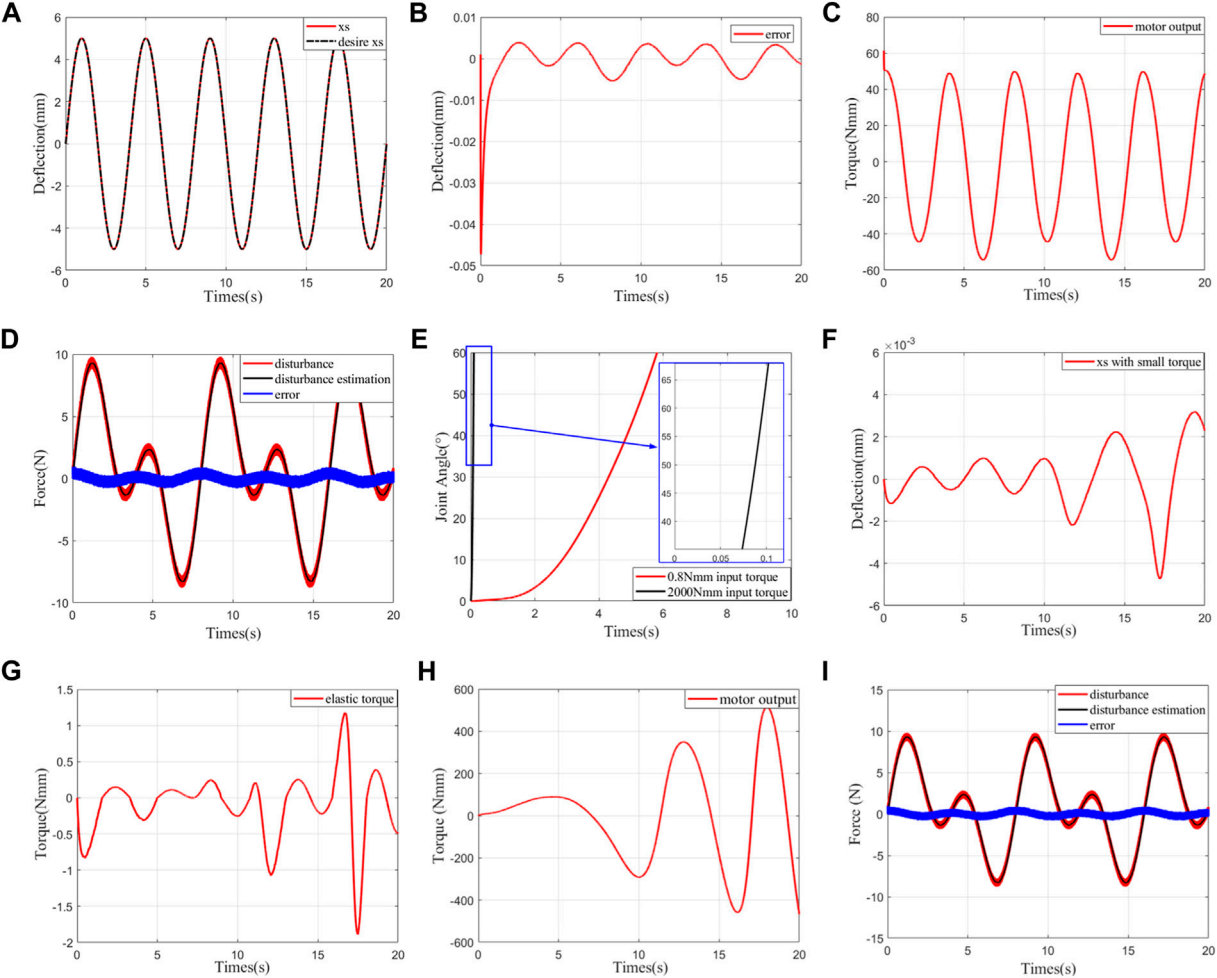
Simulation results. **(A)** Trajectory tracking of deflection at fixed output. **(B)** Error of deflection trajectory tracking at fixed output. **(C)** Motor output/control law at fixed output. **(D)** Trajectory of disturbance observation at fixed output. **(E)** Corresponding joint angles in zero-impedance control under different human–robot interaction torques. **(F)** Deflection trajectory tracking at 0.8 Nmm human–robot interaction torque under zero-impedance control. **(G)** Elastic torque $f\left(x_{s}\right)r\cos\beta$ applied to the joint/load side at 0.8 Nmm human–robot interaction torque under zero-impedance control. **(H)** Motor output/control law at 0.8 Nmm human–robot interaction torque under zero-impedance control. **(I)** Trajectory of disturbance observation at 0.8 Nmm human–robot interaction torque under zero-impedance control.

1) Simulation 1: fixed output: in this group, the main purpose is to verify the stability of the controller. To simulate the irregularity of the actual disturbance set as much as possible to verify the anti-disturbance capability of the controller, the disturbance is defined as d(t)=5⁡sin(0.5πt)+5⁡sin(0.25πt)+rand , where rand is a positive random number that is generated in each sampling period with a mean value of 0 and a variance of 1. The desired trajectory of the elastic body deformation is selected at $x_{sd}=5\sin\left(0.5\pi t\right)$ . The tuned control parameters are c=5 , $k_{0}=7.5$ , γ=1 , Δ=0.05 , and K=10 . The simulation results are shown in Figures 5A–D. The simulation shows that the tracking effect is very good, and the peak error is less than 1%. The observer can keep up with the disturbance perfectly. The disturbance error is almost generated by the random sequence rand. There is no chattering on the motor output. Thus, both the controller and observer are verified to be correct.
2) Simulation 2: zero-impedance control: in this group, the main purpose is to verify the correctness of zero-impedance control. Herein, the load side moment of inertia is selected as $J_{l}=8.9\times 10^{-3}\left(kgm^{2}\right)$ , damping is selected as 0 , d(t) and control parameters are the same as in simulation 1. The simulated human–robot interaction torque is 0.8 Nmm, which is considerably small for the human ankle, and a large torque of 2 Nm is selected for comparison. The control target is $x_{sd}=0$ . Figures 5E–I show the simulation results. From (E), it can be seen that a small input torque can still make the joint angle reach 40° within a limited time and the speed of the joint increases. (G) shows that the elastic resistance moment experienced is very small. (F) shows that the error of the deformation tracking effect is less than 0.005 mm, and the increasing error is caused by the increasing speed. These results indicate that the motor follows the movement of the load on the line. In addition, as seen from (I), the disturbance observer follows the disturbance just as it does with a fixed load. (H) shows that the theoretical controller is chatter-free. Thus, the effectiveness of zero-impedance control is verified.

### Experiment

After numerical simulation, we utilize the testing platform mentioned above to further verify the effectiveness of the established controller. The two different control model tests are implemented on the test platform. Subsequently, wearable zero-impedance control of the ankle exoskeleton was performed.

**FIGURE 6:**
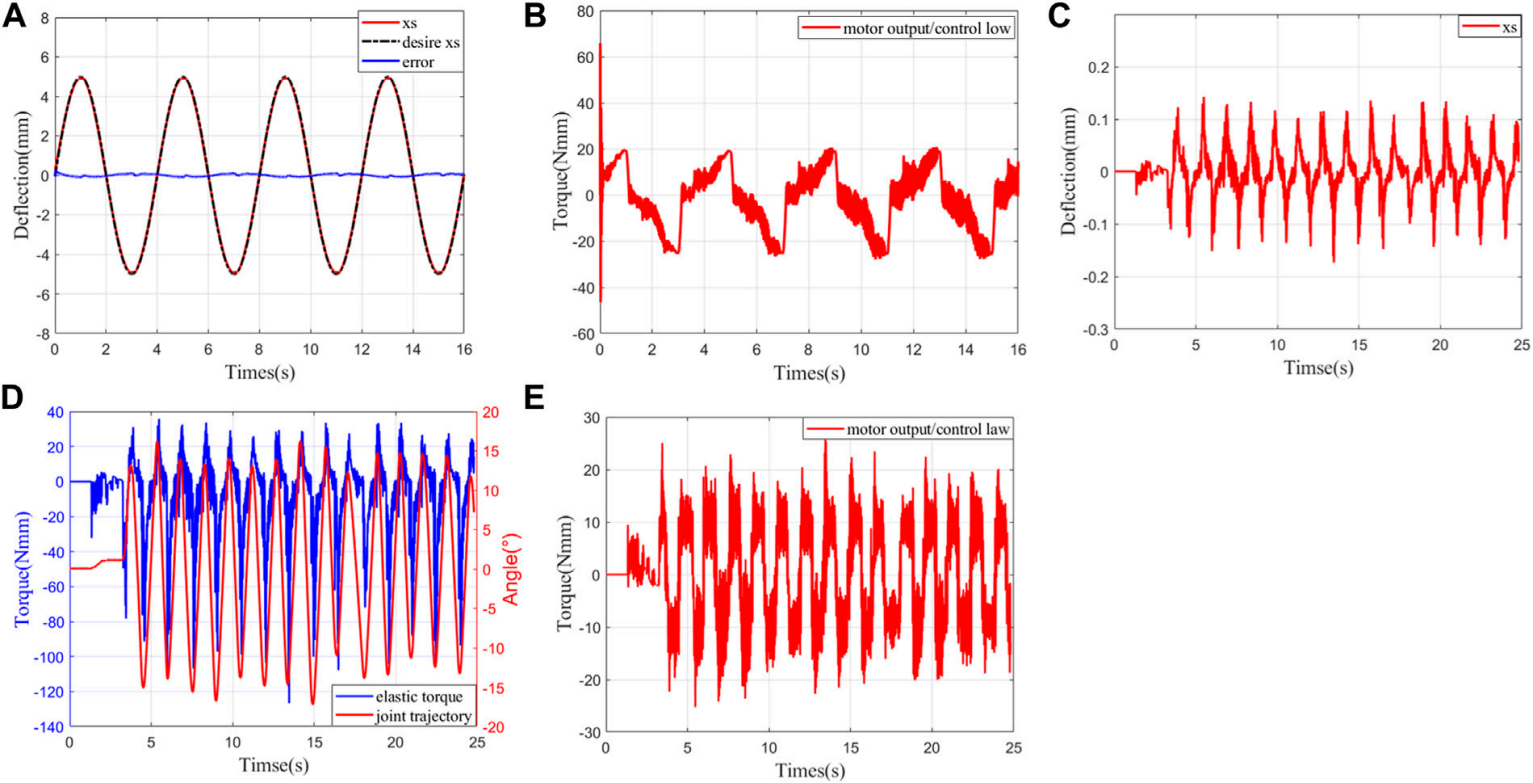
Experimental results. **(A)** Deflection trajectory tracking with fixed output. **(B)** Motor output with fixed output. **(C)** Deflection trajectory tracking under zero-impedance control. **(D)** Elastic torque and joint trajectory under zero-impedance control. **(E)** Motor output/control law under zero-impedance control.

**FIGURE 7:**
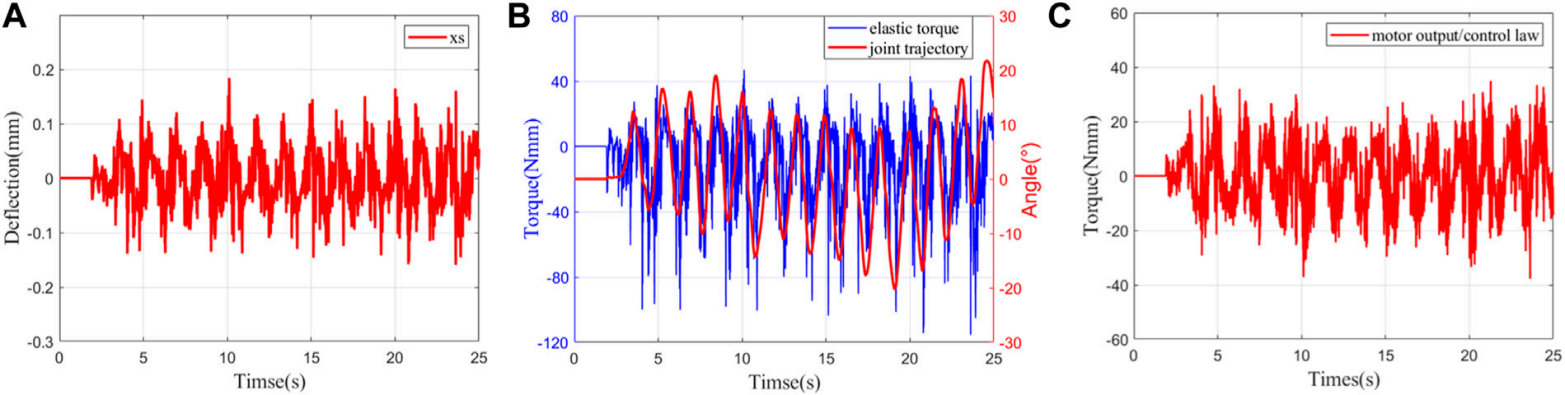
Wearing experiment results. **(A)** Deflection trajectory tracking. **(B)** Elastic torque and joint trajectory. **(C)** Motor output/control law.

1) Experiment 1: fixed output: in this group, the load side is fixed. The desired trajectory is selected as $x_{sd}=5\sin\left(0.5\pi t\right)$ . The tuned control parameters are c=10 , $k_{0}=5.55$ , γ=0.1 , Δ=0.05 , and K=14 . The experimental results are shown in Figures 6A,B. The actual peak error of deflection trajectory tracking is no more than 0.05 mm, which is close to the simulation results. The appearance of motor output chatter in (B) is caused by friction, mechanical clearance, and model error, but it is small and has no effect on the actual results. Thus, the correctness of the control algorithm is verified further. The controller can eliminate the large mechanical gap error caused by the introduction of the screw, as mentioned in *Preliminary*.
2) Experiment 2: zero-impedance control on the bench: the experimental scene is shown in Figure 1A. The external force moment is applied by hand to make the load side move according to the sinusoidal track as much as possible. The control target is $x_{sd}=0$ . The control parameters used are the same as in Experiment 1. The experimental results are shown in Figures 6C–E. We can clearly see the rather good control performance, which is also very close to the simulation results. The tracking error of impedance control is not more than 0.15 mm. The corresponding peak resistance torque (elastic torque) is approximately 100 Nmm, which is one-twentieth of the peak torque of the ankle joint when a person moves at a speed of 2.7 m/s (Witte et al., 2020). Compared to a fixed load, the motor output has a more pronounced chatter due to larger gaps, model errors and friction. However, it is acceptable that this will not affect the motion control from the trajectory of the joint angle. Consequently, the robustness of the proposed controller is demonstrated.
3) Experiment 3: wearing experiment of zero-impedance control: on the basis of Experiments 1 and 2, we control the ankle exoskeleton after wearing it to fundamentally verify the practicability of the controller. The experimental results are shown in Figure 7. Compared with Experiment 2, the tracking error is approximately the same, but the deflection and motor output chatter are further increased. Compared to Experiment 2, the tracking error values are approximately the same, but the deflection and the chattering of the motor output are further increased. This is mainly caused by the unstable binding between the human and the ankle exoskeleton, which makes the interaction force unstable. However, (B) shows that the resistance torque is still less than 100 Nmm, and the joint trajectory is also smooth, which indicates that the control is successful. The problems of large friction and gaps caused by this complex structure of LaVSA are solved. The proposed controller combined with LaVSA can be well applied to the ankle exoskeleton robot and can effectively solve the control problem during the active training of the patient during the rehabilitation process.

## Conclusion

In this study, we performed research on rehabilitation ankle exoskeleton robots combined with LaVSA for patients with neurological disorders, aiming at realizing the ‘patient-in-charge’ control method (Veneman et al., 2007). LaVSA has many advantages that make it very suitable as an ankle exoskeleton. However, these advantages of LaVSA also bring complexity at the mechanical level, larger mechanical gaps and more friction. Here, the model error caused by the gap and friction and the dynamic model of the load-side are regarded as the disturbance set. Its value is observed in real time with the disturbance observer. Most disturbance observers ideally regard the disturbance as a slow disturbance, which makes the first-order derivative of the disturbance equal to zero. However, in practice, the first-order derivative of disturbance is usually a bounded value. This usually leads to the failure of observation error convergence. Therefore, the parameter adaptation law is used to find an upper bound on the observation error. Then, corresponding compensation is made in the control law. Then, a disturbance observer-based sliding mode controller is designed. In addition, this study uses the deformation of the elastic body as an index to evaluate the low impedance property of the system. Finally, the simulation and experimental results confirm that the controller allows the patient to move freely without effort. However, because the sensor is not sufficiently good, the second-order derivative of the acquired signal is too large, which makes the control law too large. This is also a cause of chatter. Subsequent work may need to be optimized.

## Data Availability

The original contributions presented in the study are included in the article/Supplementary Material, further inquiries can be directed to the corresponding author.
